# Neuroprotective effects of mild hypothermia against traumatic brain injury by the involvement of the Nrf2/ARE pathway

**DOI:** 10.1002/brb3.2686

**Published:** 2022-07-08

**Authors:** Chaolong Yan, Jiannan Mao, Chenbei Yao, Yang Liu, Huiying Yan, Wei Jin

**Affiliations:** ^1^ Department of Neurosurgery, Nanjing Drum Tower Hospital Clinical College of Nanjing Medical University Nanjing China; ^2^ Department of Neurosurgery, Nanjing Drum Tower Hospital The Affiliated Hospital of Nanjing University Medical School Nanjing China; ^3^ Department of Neurosurgery, Zhongshan Hospital The Affiliated Hospital of Fudan University Shanghai China

**Keywords:** mild hypothermia, Nrf2, oxidative stress, traumatic brain injury (TBI)

## Abstract

**Background:**

Traumatic brain injury (TBI) is the leading cause of death and disability worldwide. Mild hypothermia (32–35°C) has been found to show neuroprotective effects against TBI. However, the specific mechanism is still elusive. In the current study, we explored the relationship between oxidative damage after TBI and treatment with mild hypothermia as well as the underlying molecular mechanisms.

**Methods:**

We used the closed cortex injury model to perform the brain injury and a temperature monitoring and control system to regulate the body temperature of mice after injury. Adult male C57BL/6 mice were adopted in this study and divided into four experimental groups. Tissue samples were harvested 24 h after injury.

**Results:**

First, our results showed that treatment with mild hypothermia significantly improved neurobehavioral dysfunction and alleviated brain edema after TBI. Moreover, treatment with mild hypothermia enhanced the activity of the antioxidant enzymes superoxide dismutase and glutathione peroxidase and reduced the accumulation of lipid peroxidation malondialdehyde. Importantly, the expression and activation of the nuclear factor erythroid 2‐related factor 2‐antioxidant response element (Nrf2‐ARE) pathway were upregulated by mild hypothermia after TBI. Finally, treatment with hypothermia significantly decreased the cell apoptosis induced by TBI.

**Conclusion:**

Our results showed that the protective effects of mild hypothermia after TBI may be achieved by the upregulation of the Nrf2‐ARE pathway and revealed Nrf2 as a potentially important target to improve the prognosis of TBI.

## Graphical abstract

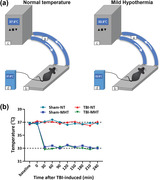

## 1 INTRODUCTION

Traumatic brain injury (TBI) is a major cause of young people death around the world, leaving a variety of physical disability and psychological sequelae (Amyot et al., 2018; Lucke‐Wold, Smith, et al., 2015). Although considerable studies have been performed thus far, the clinical prognosis remains poor. A large amount of evidence has shown that secondary brain injury, such as oxidative stress, plays a vital role in the pathological mechanisms of TBI (Kumar Sahel et al., 2019; Ladak et al., 2019). Previous studies have reported that reactive oxygen species (ROS) are overproduced in brain tissue following TBI. The formation of ROS can trigger subsequent oxidative stress‐related molecular damage, such as protein oxidation, lipid peroxidation, and DNA damage, ultimately resulting in cell apoptosis (Di Pietro et al., 2020). This close relationship between oxidative stress and the mechanisms of TBI makes antioxidant therapies the focus of research on the treatments of TBI.

Mild hypothermia, generally referring to a temperature between 33°C and 35°C, has been proven to be an efficient technique to alleviate neuronal injury in central system injuries, including TBI, spinal cord injury, and ischemic or hemorrhage strokes (Ahmad et al., 2014; Sheng et al., 2012; Song, Wang et al., 2020; Wu & Grotta, 2013). In recent years, the literature has reported that mild hypothermia (MHT) can reduce cerebral edema and decrease intracranial pressure in clinical practice, thereby protecting brain tissues (Andrews et al., 2015; Jiang et al., 2006). The neuroprotective effects of MHT have also been demonstrated in preclinical research on experimental animals by alleviating neurological dysfunction, attenuating brain edema, and reducing apoptosis, among other benefits (H.‐B. Zhang et al., 2017; C. C. Zhao et al., 2017). However, it is still not clear whether the nuclear factor erythroid 2‐related factor 2 (Nrf2)‐mediated antioxidative system is involved in the treatment of MHT after TBI.

Nrf2, a key transcription factor, plays an important role against oxidative stress. Under physiological conditions, Nrf2 is mainly located in the cytoplasm and binds with its adapter protein Kelch‐like ECH associated protein 1 (Keap1) as a complex, finally facilitating the ubiquitination of Nrf2 (Cores et al., 2021). Upon exposure to oxidative stress, Nrf2 dissociates from Keap1 and translocates into the nucleus to interact with the antioxidant response element (ARE), which regulates the transcription of antioxidant genes such as heme oxygenase‐1 (HO‐1) and NAD(P)H:quinone oxidoreductase‐1 (NQO‐1) (Francis et al., 2019). Our previous studies confirmed the protective role of the Nrf2‐ARE signaling pathway in TBI, and knockout of the Nrf2 gene also aggravated oxidative injury and apoptosis in a TBI mouse model (Dai et al., 2018; Jin et al., 2011; Lu et al., 2015). The present study aimed to investigate the potential protective effect of MHT on the TBI mouse model and explore whether the therapeutic benefits of MHT are related to the activation of the Nrf2‐ARE signaling pathway and subsequently alleviate oxidative stress damage.

## 2 METHODS

### 2.1 Animals and groups design

All experimental procedures were in accordance with the guidelines of the National Institutes of Health on the care and use of animals and approved by the Institutional Animal Care and Use Committee at Nanjing Drum Tower Hospital. Adult male C57BL/6 mice (8–10 weeks old, 20–25 g) were housed in a humidity‐controlled room (25 ± 1°C, 12‐h light/dark cycle) and were provided free access to water and food.

A total of 104 mice were randomly divided into four groups: the sham with normothermia group (Sham‐NT), the sham with mild hypothermia group (Sham‐MHT), the TBI with normothermia group (TBI‐NT), and the TBI with mild hypothermia group (TBI‐MHT). From each group, eight animals were randomly selected for behavioral evaluation (modified Neurological Severity Score [mNSS] and rotarod tests), and six animals were sacrificed for molecular detection and histological evaluation.

### 2.2 Traumatic brain injury

A closed cortex injury model was used to induce TBI as described in our previous study (Jin et al., 2011; C. Yan et al., 2020). Animals were anesthetized by intraperitoneal injection with 10% chloral hydrate (0.5 g/kg). A midline incision and a 3.5 mm craniotomy were conducted over the right parietal cortex. TBI was induced by a 40 g weight drop from a 10 cm height along a stainless‐steel string to strike a hit pillar located at the cortex. After recovery from anesthetization, animals were returned to their cages and housed at 25 ± 1°C. The sham mice were subjected to identical operations without injury.

### 2.3 Temperature monitoring and control system

**FIGURE 1:**
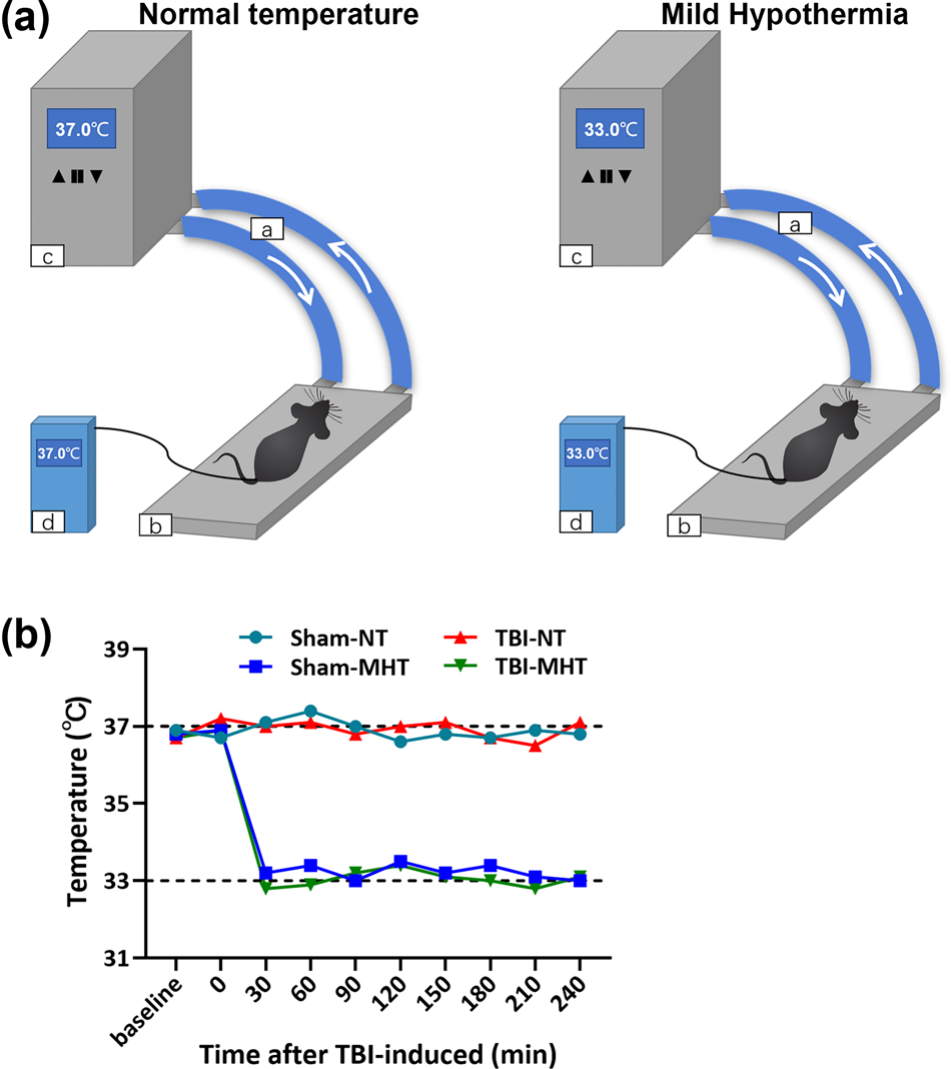
Schematic diagram of the temperature monitoring and control system and the monitored temperature curves. (a) Schematic diagram of the temperature monitoring and control system. (a) Water flow, (b) platform regulating the body temperature of mice, (c) temperature controller regulating the temperature of the flowing water, and (d) temperature monitor showing the real‐time temperature of mice. (b) The temperature curves of different groups based on the baseline and monitored temperatures

We used a temperature monitoring and control system (Zhongshidichuang Science and Technology, Beijing, China) to regulate the body temperature of the experimental animals. As shown in Figure 1a, animals were placed on the platform, and the body temperature was regulated by the water flow from the controller, the role of which was to heat or cool the water flow. Rectal thermometers were used to monitor the temperature of the mice. In our experiments, the temperature manipulation was conducted immediately after surgery and maintained for 4 h. The body temperatures of mice in the Sham‐NT and TBI‐NT groups were maintained at 33°C, and those in the Sham‐MHT and TBI‐MHT groups were maintained at 37°C. During the thermoregulation periods, the temperatures of the mice were recorded every 30 min. Then, all mice were allowed to return to their baseline temperatures. The temperature curve was plotted according to the baseline temperatures before TBI surgery, the monitored temperatures during thermoregulation, and the real‐time temperatures before sacrifice at 24 h after TBI, as shown in Figure 1b.

### 2.4 Tissue preparation

Mice were euthanized at 24 h post‐TBI by deep anesthetization with sodium pentobarbital (100 mg/kg) and perfused via the left cardiac ventricle with cold phosphate‐buffered saline (PBS, 4°C). Brains were dissected on ice, and the tissues around the injury site were collected and stored in liquid nitrogen for further measurements for the analysis of western blotting, qPCR, malondialdehyde (MDA), superoxide dismutase (SOD), and glutathione peroxidase (GPX). For immunofluorescence analysis, the brains were immersed in 4% buffered paraformaldehyde (4◦C) overnight and sectioned at 4 εm thickness with a microtome for further study.

### 2.5 Measurement of brain edema

The wet/dry weight method was used to measure brain edema after TBI as previously described (Jin et al., 2011; C. Yan et al., 2020). After deep anesthetization with 10% chloral hydrate (0.5 g/kg), the mice were sacrificed 24 h after TBI. The brain tissue was removed and immediately weighed to obtain the wet weight (WW). Then, the samples were dried for 24 h at 110°C and reweighted to obtain the dry weight (DW). Brain water content was calculated as [(WW – DW) × 100%]/WW.

### 2.6 Neurological deficit evaluation

Neurological deficits were evaluated with the mNSS and rotarod tests following our previous work (C. Yan et al., 2020). The mNSS tests were performed to evaluate the overall neurological deficits, including tests about the motor, sensory, and reflex of animals. The score of the mNSS tests ranged from 0 to 18 (0 = normal; 18 = the maximal deficit). The higher the score, the more severe the neurological dysfunction was. The motor coordination and balance ability of the animals were measured by rotarod tests. Mice were placed on a rotarod apparatus with an acceleration of 10 rpm/min (RWD Life Science, Shenzhen, China). When mice fell off the rotating rod, the time was recorded automatically. This process was repeated three times to calculate the average latency time.

### 2.7 Western blotting

Western blotting was conducted as previously described (C. Yan et al., 2020; H. Yan et al., 2015). The cortex tissues were homogenized in lysis buffer (Thermo Fisher Scientific, Waltham, MA) and centrifuged at 12,000 × g for 15 min at 4°C. The supernatant was collected as the total protein. The protein concentration was measured by a BCA assay kit (Thermo Fisher Scientific). Equivalent amounts of protein from each sample were loaded and electrophoretically separated on a 10–15% SDS‐PAGE gel and then transferred onto polyvinylidene fluoride membranes (Millipore, Darmstadt, Germany). The membranes were blocked with 5% skim milk‐Tris‐buffered saline containing Tween 20 (TBST) for 1 h at room temperature and subsequently incubated with the primary antibodies (anti‐Nrf2, 1:200, Santa Cruz Biotechnology, sc‐365949, USA) at 4°C overnight. After washing four times (5 min/wash) with TBST, the membranes were incubated with horseradish peroxidase‐combined secondary antibodies (1:10,000; Beyotime, Shanghai, China) for 1 h at room temperature. After incubation and washing four times again, the bands were visualized and developed by an enhanced chemiluminescence kit (Thermo Fisher Scientific). The densitometric analysis of the bands was performed by using ImageJ software (NIH, USA).

### 2.8 Quantitative PCR

Total RNA was extracted from tissues from TBI mice according to the manufacturer's instructions (Vazyme, Nanjing, China). RNA samples were then reverse transcribed to cDNA by using a reverse transcription (RT) reagent kit (Thermo Fisher Scientific). Quantitative PCR (q‐PCR) was carried out following the PowerUp SYBR Green Master Mix kit (Thermo Fisher Scientific). The reaction system was a 20‐εl mixture in a 96‐well block containing 10 εl SYBR Green, 0.4 εl upstream primer, 0.4 εl downstream primer, 2 εl cDNA, and 7.2 εl RNase‐free water (C. Yan et al., 2020). The primers used in this work were as follows: NQO1: forward: 5′‐CATTCTGAAAGGCTGGTTTGA; reverse: 5′‐CTAGCTTTGATCTGGTTGTCAG‐3′; HO‐1: forward: 5′‐ATCGTGCTCGCATGAACACT‐3′; reverse: 5′‐CCAACACTGCATTTACATGGC‐3′; β‐actin: forward: 5′‐AGTGTGACGTTGACATCCGTA‐3′; reverse: 5′‐GCCAGAGCAGTAATCTCCTTCT‐3′ (Jin et al., 2009). The quantification of target genes in each tissue was normalized to actin mRNA levels and calculated by the 2^−ΔΔCt^ method.

### 2.9 Detection of MDA, SOD, and GPX

The MDA content in each sample was detected by an MDA assay kit according to the manufacturer's instructions (Nanjing Jiancheng Biochemistry Co., Nanjing, China). The SOD and GPX activities from tissue samples were examined by a detection kit (Beyotime) in accordance with the manufacturer's instructions. The concentration of total protein was measured by a BCA assay kit (Thermo Fisher Scientific).

### 2.10 Terminal deoxynucleotidyl transferase biotin‐dUTP nick end labeling

The 4% paraformaldehyde‐fixed, paraffin‐embedded tissue samples were dehydrated in 30% saccharose phosphate‐buffered saline (PBS) for 2 days and then sectioned at 4 εm thickness with a microtome. According to our previous work (C. Yan et al., 2020; H. Yan et al., 2015), terminal deoxynucleotidyl transferase (TdT) dUTP nick‐end labeling (TUNEL) staining was conducted following the manufacturer's instructions (Beyotime). The sections were incubated with the TUNEL reaction mixture at 37°C for 2 h and counterstained with 4′,6‐diamidino‐2‐phenylindole (DAPI, Beyotime). The TUNEL‐positive cells were identified, counted, and analyzed to determine the apoptotic index, which was calculated as the average percentage of TUNEL‐positive cells in four cortical microscopic fields (×400) from each section. Three sections from each animal were considered.

## 3 RESULTS

### 3.1 MHT attenuated neurological deficits after TBI

**FIGURE 2:**
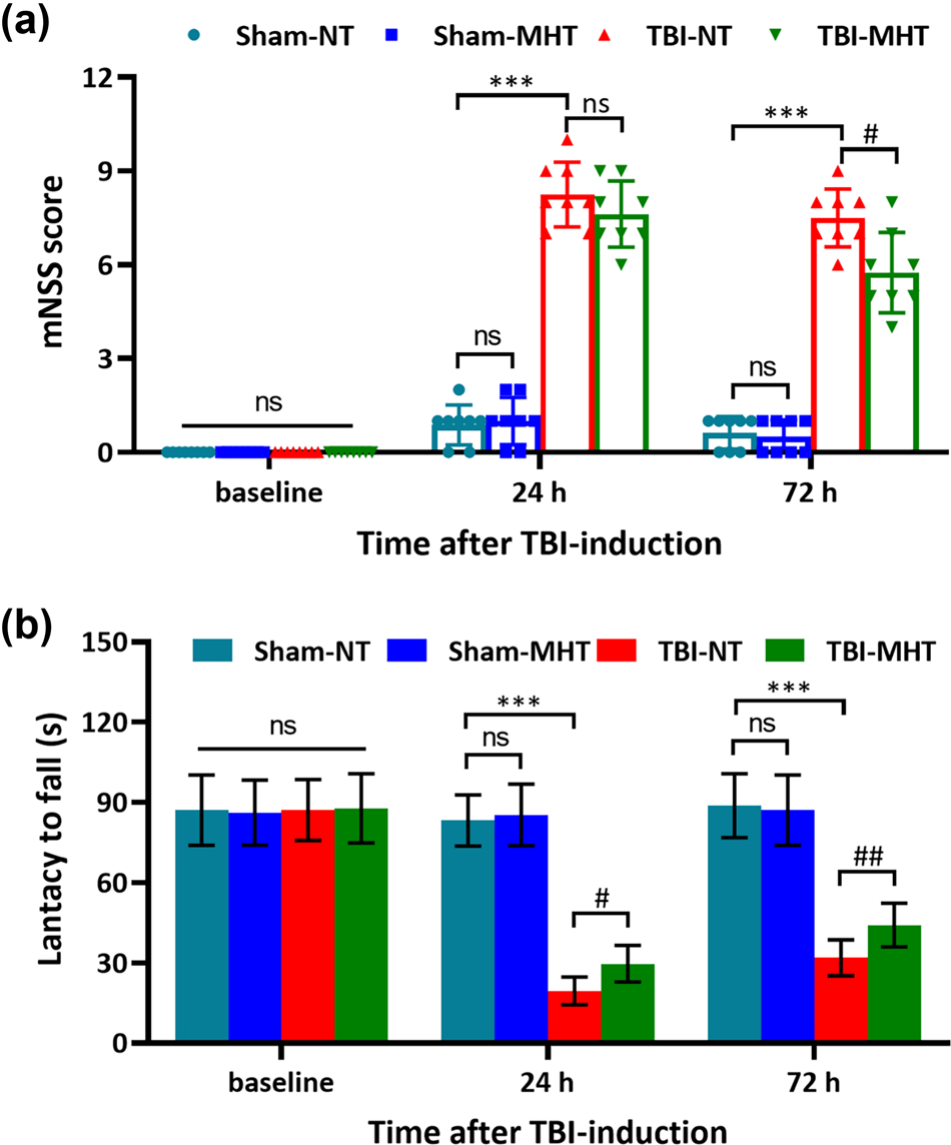
Treatment with mild hypothermia attenuated the neurological dysfunctions induced by traumatic brain injury (TBI). Neurobehavioral performance was assessed by modified Neurological Severity Score (mNSS) (a) tests and rotarod tests (b), both before injury modeling and at 24 and 72 h after TBI induction. *n* = 8/group. Data are presented as the mean ± SEM. ^***^ *p* < .001, ^#^ *p* < .05, ^##^ *p* < .01, ^ns^ *p* > .05

The mNSS tests and rotarod tests were performed to measure neurological dysfunctions at 24 h and 72 h after TBI. All animals underwent a training trial to exclude the individual differences among various (Figure 2a,b). At 24 h and 72 h post‐TBI, the mNSS scores in the TBI‐NT group were significantly increased compared with those in the Sham‐NT group, which indicates marked neurobehavioral loss by TBI induction. After treatment with MHT, the scores in the TBI‐MHT group were decreased at 72 h post‐TBI compared with those in the TBI‐NT group. However, there was no significant difference between the Sham‐NT and Sham‐MHT groups (Figure 2a). Similarly, the coordination and balance abilities evaluated by rotarod tests were remarkably impaired in TBI mice, and MHT treatment prolonged the latency time of mice on the Rota‐rod, indicating the improvement of the coordination and balance abilities (Figure 2b).

### 3.2 MHT increased the activation of the Nrf2‐ARE pathway

**FIGURE 3:**
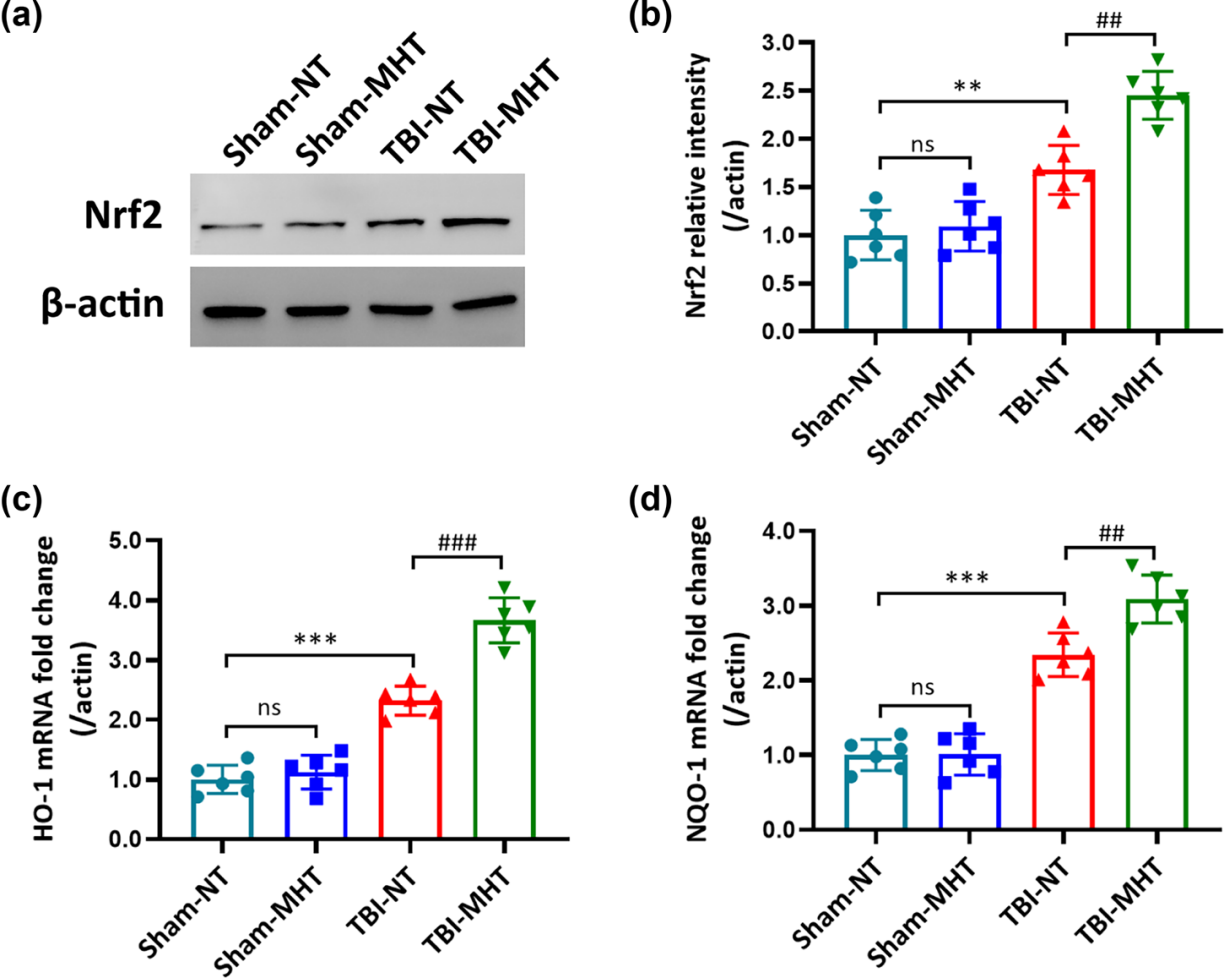
Treatment with mild hypothermia upregulated nuclear factor erythroid 2‐related factor 2‐antioxidant response element (Nrf2‐ARE) pathway expression and activation after traumatic brain injury (TBI). (a) Representative immunoblot bands and (b) quantitative analysis of Nrf2 at the protein level at 24 h after TBI. Real‐time quantitative PCR analysis of the downstream factors of the Nrf2 signaling pathway and the mRNA levels of heme oxygenase‐1 (HO‐1) (c) and NAD(P)H: quinone oxidoreductase‐1 (NQO‐1) (d). *n* = 6/group. Data are presented as the mean ± SEM. ^**^ *p* < .01, ^***^ *p* < .001, ^##^ *p* < .01, ^###^ *p* < .001, ^ns^ *p* > .05

To determine the activation of the Nrf2‐ARE pathway after TBI with or without MHT, we detected the protein expression of nuclear Nrf2 by western blotting. As shown in the results, the expression of nuclear Nrf2 was significantly increased at 24 h after TBI. Compared with the TBI‐NT group, the protein level of nuclear Nrf2 was further upregulated in the TBI‐MHT group (Figure 3a,b). Furthermore, we also examined the mRNA levels of HO‐1 and NQO‐1 by qPCR, downstream factors of the Nrf2‐ARE pathway, which are involved in the resistance to oxidative stress. In comparison with the TBI‐NT group, treatment with MHT likewise increased the expression of HO‐1 and NQO‐1 at mRNA levels (Figure 3c,d). However, no significant difference was found in the Sham‐NT and Sham‐MHT groups.

### 3.3 MHT reduced oxidative stress following TBI

**FIGURE 4:**
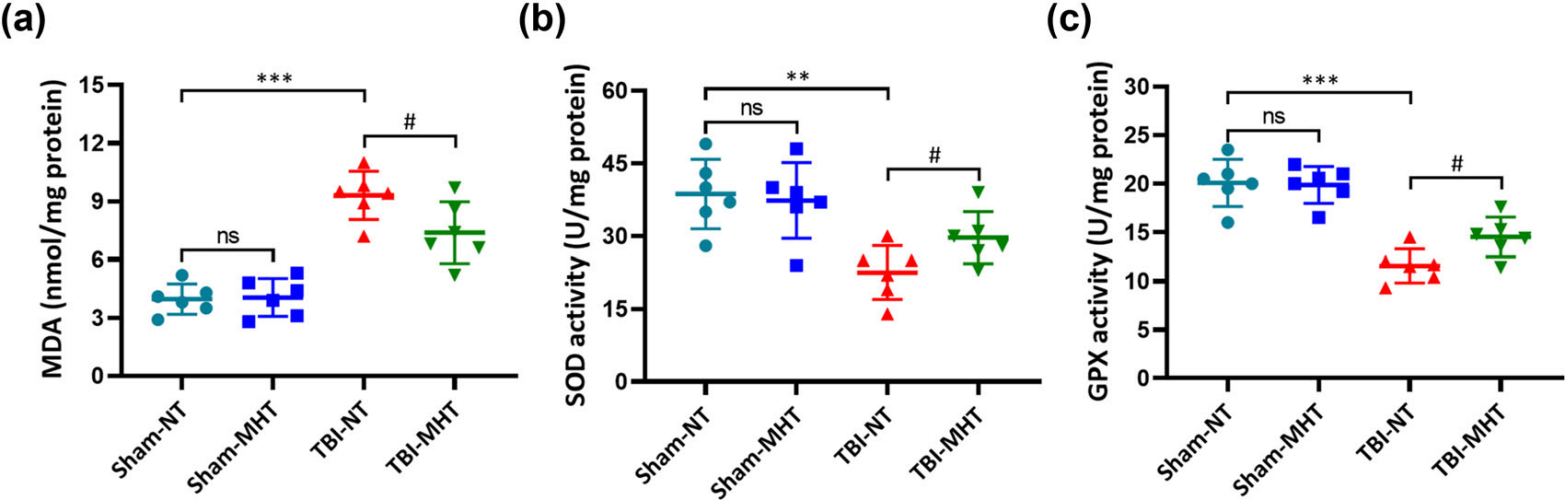
Treatment with mild hypothermia alleviated oxidative stress after traumatic brain injury (TBI). The malondialdehyde (MDA) (a), superoxide dismutase (SOD) (b), and glutathione peroxidase (GPX) (c) contents were detected by assay kits. *n* = 6/group. Data are presented as the mean ± SEM. ^**^ *p* < .01, ^***^ *p* < .001, ^#^ *p* < .05, ^ns^ *p* > .05

The activation of the Nrf2‐ARE pathway plays a vital role in antioxidative stress by reducing antioxidative production and increasing the activity of antioxidative enzymes. To explore the effects of the activation of the Nrf2‐ARE pathway, we detected the content of MDA and the activation of SOD and GPX in the brain tissues. MDA is one of the lipid peroxides, representing the level of lipid peroxidation. SOD and GPX are antioxidant enzymes that eliminate the metabolites of free radicals. As shown in the results, the level of MDA was increased in the TBI‐NT group compared with the Sham‐NT group. Treatment with MHT reduced the MDA content in injured brain tissues (Figure 4a). In contrast, the activities of SOD and GPX decreased after TBI induction and were upregulated in the MHT‐treated TBI group (Figure 4b,c).

### 3.4 MHT post‐TBI alleviates brain edema

**FIGURE 5:**
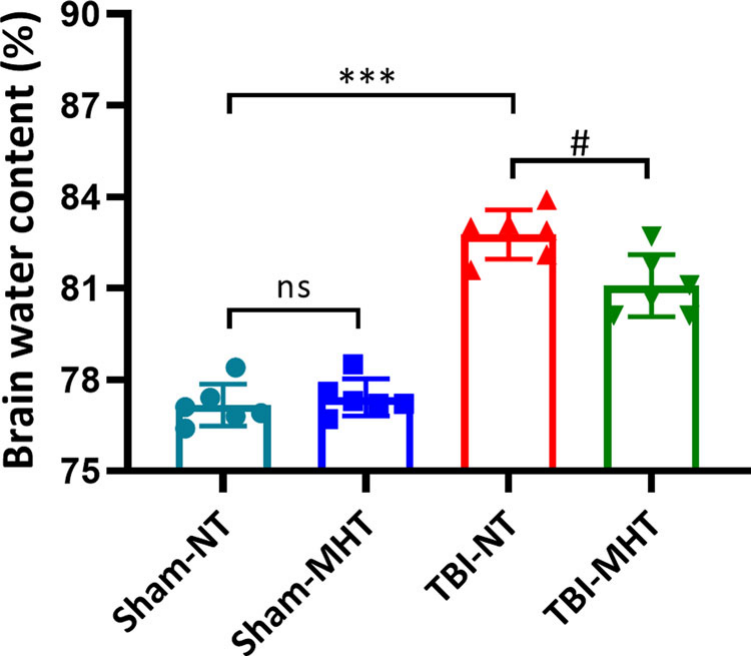
Treatment with mild hypothermia reduced the water contents of brain tissues after traumatic brain injury (TBI) induction. Brain edema was measured by the dry/wet method. Mild hypothermia significantly alleviated the brain edema of injured tissues. *n* = 6/group. Data are presented as the mean ± SEM. ^***^ *p* < .001, ^#^ *p* < .05, ^ns^ *p* > .05

To evaluate brain edema after TBI, the dry/wet weight method was adopted. After drying and reweighing, the brain water content in the Sham‐NT and Sham‐MHT groups showed no significant difference. TBI induced a higher rate of brain water content than that in the Sham‐NT group. Compared with the TBI‐NT group, the TBI‐MHT group had a decline in brain water content (Figure 5).

### 3.5 MHT decreased apoptosis after TBI

**FIGURE 6:**
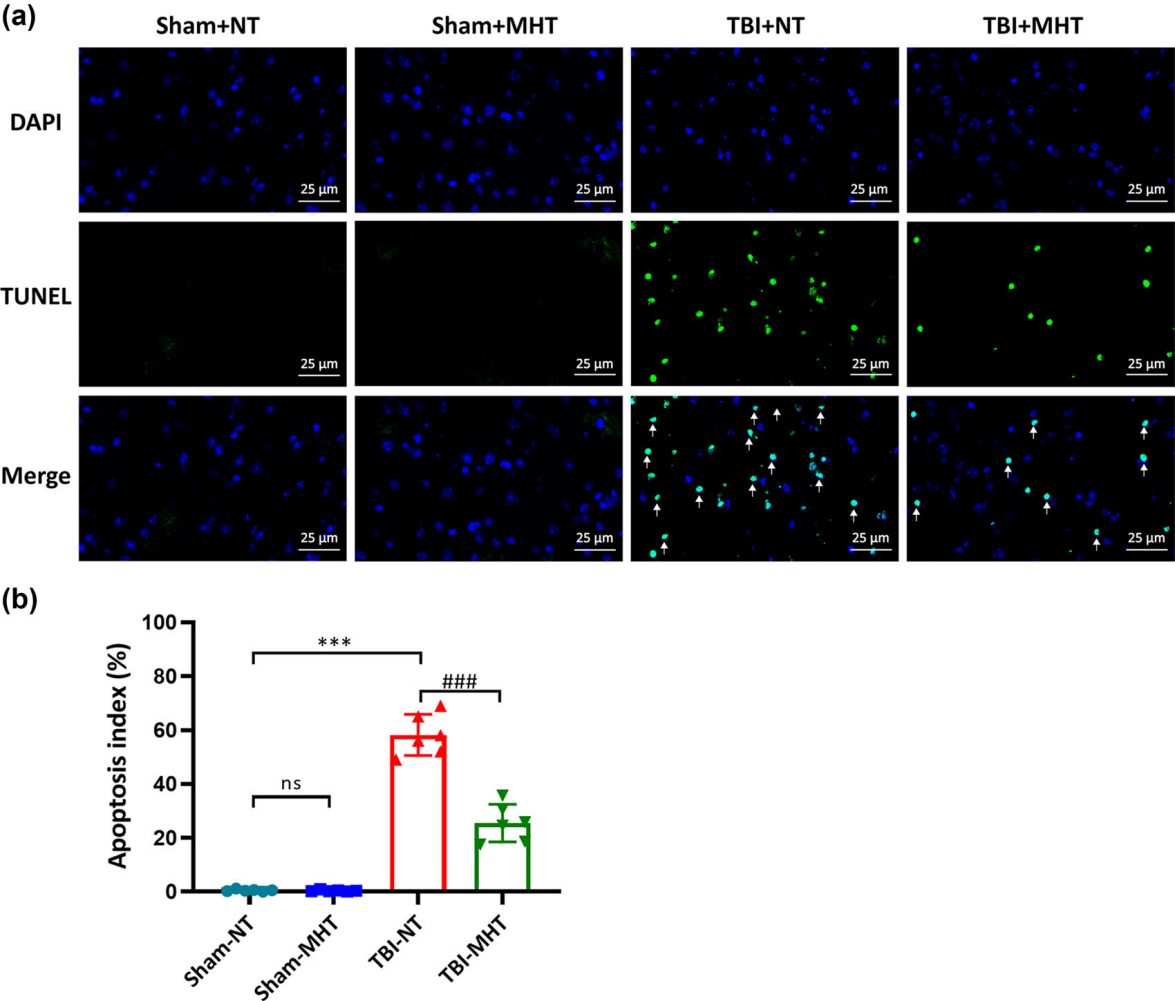
Treatment with mild hypothermia decreased cell apoptosis after traumatic brain injury (TBI). Terminal deoxynucleotidyl transferase biotin‐dUTP nick end labeling (TUNEL) assay was used to evaluate the levels of apoptosis in different groups. (a) Representative images of TUNEL staining from each group. White arrows indicate TUNEL‐positive cells. (b) The apoptotic index was calculated at 24 h after TBI. The percentage of apoptotic cells was significantly decreased by treatment with mild hypothermia after TBI. *n* = 6/group. Data are presented as the mean ± SEM. ^***^ *p* < .001, ^###^ *p* < .001

Oxidative stress and brain edema have a fatal effect on cells, especially on apoptosis. To explore whether MHT could further affect apoptosis after brain injury, TUNEL staining was employed to detect apoptotic cells, and the apoptotic index was calculated to measure neural death. In the Sham‐NT and Sham‐MHT groups, few TUNEL‐positive cells were labeled. More TUNEL‐positive cells were found in the TBI‐NT group than in the Sham‐NT group. Moreover, treatment with MHT after TBI reduced the number of apoptotic cells compared with the normal temperature group (Figure 6a,b).

## 4 DISCUCTION

TBI is a complex and severe disease with multiple pathological processes, such as the oxidative response, inflammatory response, and cell apoptosis (J. Zhao et al., 2020). In recent years, an increasing number of studies have shown that MHT has a protective effect against TBI. However, the specific mechanism is still unclear, especially the relationship between MHT and the oxidative stress response after TBI. In our study, we found that the Nrf2‐ARE‐mediated antioxidative pathway was involved in the neuroprotective effects of MHT on TBI. First, we showed that treatment with MHT increased the activation of the Nrf2‐ARE pathway following TBI and significantly improved neurobehavioral performance compared with normothermia. In addition, treatment with MHT enhanced the activity of the antioxidant enzymes SOD and GPX and reduced the content of the lipid peroxidation product MDA in injured brain tissue. Finally, we also found that the brain edema and cell apoptosis induced by TBI were also relieved in the MHT group.

Oxidative stress damage has been recognized as an important player in secondary brain injury after TBI, which is characterized by the overproduction of ROS and the accumulation of lipid hydroperoxides (Hall et al., 2019). Under physiological conditions, antioxidant substances can remove certain ROS, making the endogenous antioxidant system and the production of ROS in a dynamic equilibrium state (Mohammed et al., 2020). However, under the pathological condition of tissue damage, for example, TBI, there is an excessive release of free radicals, which causes cytotoxic responses such as DNA damage, lipid peroxidation, mitochondrial dysfunction, and cell apoptosis (Khatri et al., 2018; Lamade et al., 2020; Mendes Arent et al., 2014). Based on this, some antioxidative drugs have been studied to alleviate oxidative stress, showing neuroprotective effects in animal models, such as salubrinal, melatonin, carveol, and carvacrol (Ali et al., 2020; Logsdon et al., 2016; Lucke‐Wold, Naser, et al., 2015; Malik et al., 2020; Naeem et al., 2021). In the present study, the activities of the antioxidative enzymes SOD and GPX were significantly decreased after TBI induction. As a result, the level of the lipid peroxide MDA was elevated to some extent in TBI mice compared with the sham group. In addition, a large number of apoptotic cells were observed in the injured brain tissues. These phenomena supported that the occurrence of TBI induced the overactivation of oxidative stress and the related cell death cascade.

MHT has long been considered to have neuroprotective effects on TBI in some small clinical trials and various experimental models (Gao et al., 2021; Song et al., 2020; C.‐F. Wang et al., 2019). In 2001, a multicenter study explored the difference in prognosis between normal temperature and hypothermia TBI patients and reported that individuals who were already hypothermic at admission could achieve better outcomes (Clifton et al., 2002, 2001). It directly enhanced awareness of the need to achieve the hypothermic state as soon as possible for patients with TBI to improve therapy efficiency. Over the past decades, although there have been an increasing number of clinical studies on the therapeutic effects of MHT, no consistent conclusions have been reached (Shaefi et al., 2016). Meanwhile, fundamental research on the mechanisms of the influence of MHT on TBI has also been moving on. Pharmacological hypothermia induced by HPI201, a neurotensin receptor agonist, reduced the inflammatory response and improved blood–brain barrier integrity after TBI (Gu et al., 2015). Endoplasmic reticulum stress‐induced apoptosis has also been reported to be reduced by MHT in a mouse TBI model (C.‐F. Wang et al., 2019). However, the relationship between oxidative stress and MHT following TBI is still unknown. Our results showed that MHT could significantly improve the activities of the antioxidative enzymes SOD and GPX and decrease the level of the lipid peroxide MDA. Moreover, the apoptotic index showed a downward trend after treatment with MHT in TBI mice. In addition, we found that brain edema and neurological function after TBI were significantly improved in the MHT group compared with the normal‐thermic group. The treatment of MHT after TBI significantly alleviated the oxidative damage in experimental mice.

Nrf2 is a basic leucine zipper transcription factor that plays a vital role in regulating cellular responses to oxidative stress (He et al., 2019; Lei et al., 2020; L. Zhang & Wang, 2018). Accumulating evidence has suggested that Nrf2 is activated after TBI to exert antioxidative effects (Li et al., 2021; H. Wang et al., 2020). Under basal physiological conditions, Nrf2 is sequestered in the cytoplasm and is degraded by binding to its suppressor Keap1 (H. Wang et al., 2020). However, to combat the stress of tissue injury, Nrf2 dissociates from Keap1 and translocates into the nucleus, finally activating the Nrf2‐ARE signaling pathway and releasing antioxidative factors (Shen et al., 2019; H. Wang et al., 2020). In the present study, we found that the expression of Nrf2 was significantly increased after MHT treatment. Moreover, the levels of the antioxidative factors HO‐1 and NQO‐1, which are activated by the Nrf2‐ARE signaling pathway, were also upregulated in the injured brain tissues. The activities of the antioxidative enzymes SOD and GPX were improved to reduce the accumulation of MDA, significantly alleviating the oxidative damage after TBI. Meanwhile, treatment with MHT following TBI reduced the number of apoptotic cells in the injured brain, reducing the number of neurons suffering from secondary injury. These findings suggest that by treatment with MHT, the activation of the Nrf2‐ARE signaling pathway was upregulated to increase the antioxidative effects and exert neuroprotective effects after TBI.

Collectively, the current study demonstrates that MHT therapy after TBI could increase the activation of the Nrf2‐ARE signaling pathway to alleviate oxidative damage and reduce cell apoptosis in injured brain tissues. As a result, brain edema and neurobehavioral dysfunctions induced by TBI were remarkably improved by treatment with MHT.

## 5 CONCLUSION

In summary, we provide evidence that MHT therapy could exert antioxidative and antiapoptotic bioactivities in a TBI mouse model. The underlying molecular mechanisms of these beneficial effects were related to the Nrf2‐ARE signaling pathway mediating the regulation of oxidative stress and apoptosis. Our experimental results provide a view to explain the neuroprotective effects of MHT by upregulating the activation of the Nrf2‐ARE signaling pathway‐mediated antioxidative effects.

## CONFLICT OF INTEREST

The authors declare no conflict of interest.

## AUTHOR CONTRIBUTIONS

Chaolong Yan designed the project and wrote the manuscript. Jiannan Mao participated in the TBI model and analyzed the data of the animal studies. Chaolong Yan performed the western blotting and RT–PCR. Yang Liu performed TUNEL staining. Huiying Yan and Wei Jin contributed to conceiving and provided critical revisions. All authors checked and approved the final manuscript.

### PEER REVIEW

The peer review history for this article is available at: https://publons.com/publon/10.1002/brb3.2686.

## Data Availability

The raw data supporting the conclusions of this article will be made available by the authors, without undue reservation.
